# Supplementary material for: Understanding the heterogeneous performance of variant effect predictors across human protein-coding genes
Source: Sci Rep. 2024 Oct 30;14:26114. doi: 10.1038/s41598-024-76202-6 (PMC11526010; doi:10.1038/s41598-024-76202-6)
Supplement: Supplementary file 1 — Supplementary Material 1 [file 41598_2024_76202_MOESM1_ESM.docx]

**Supplemental Material for:**

**Understanding the heterogeneous performance of variant effect predictors across human protein-coding genes**

Mohamed Fawzy and Joseph A. Marsh*

*MRC Human Genetics Unit, Institute of Genetics and Cancer, University of Edinburgh, Edinburgh, UK*


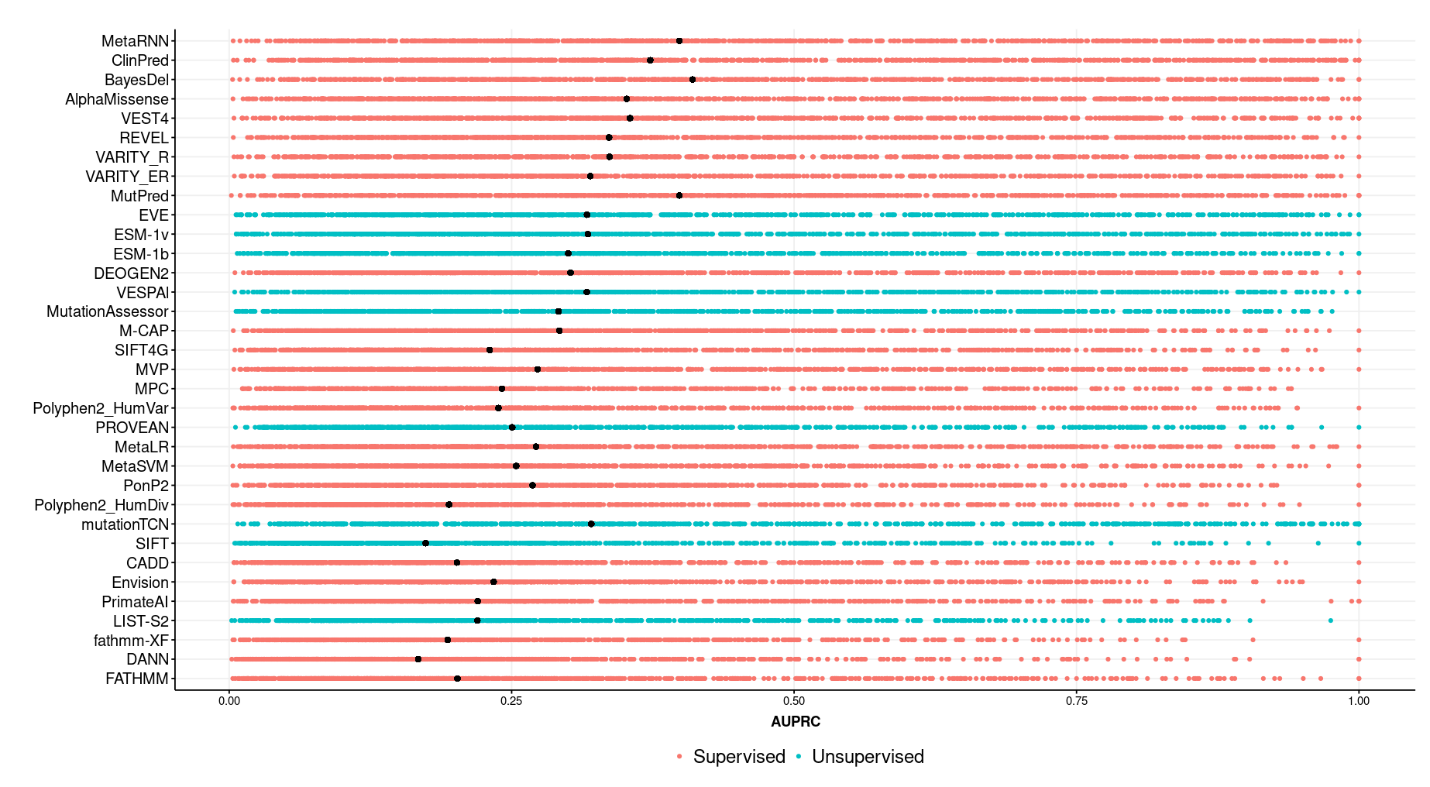


**Figure S1**: **Distribution of area under the precision-recall curve (AUPRC) values for different VEPs.** This figure is the same as Figure 1 but using AUPRC instead of AUROC.

**Table S1: Description of features used in the random forest models.**

| **Feature** | **Description** | **Source** |
| --- | --- | --- |
| transcript_count | The count of Alternatively Spliced Gene Transcripts | |
| ntissues | The number of tissues where the gene is actively expressed | Human Protein Atlas |
| nTPM | The normalized Transcript per Million quantity for gene Expression | Human Protein Atlas |
| obs_mis | Number of observed missense variants in transcript | gnomAD genes Constraints |
| exp_mis | Number of expected missense variants in transcript | |
| oe_mis | Observed over expected ratio for missense variants in transcript (obs_mis divided by exp_mis) |  |
| possible_mis | Number of possible missense variants in transcript | |
| obs_lof | Number of observed predicted loss-of-function (pLoF) variants in transcript | |
| mu_lof | Mutation rate summed across all possible pLoF variants in transcript | |
| possible_lof | Number of possible pLoF variants in transcript | |
| pRec | Probability that transcript falls into distribution of recessive genes (~46% o/e pLoF ratio; computed from gnomAD data) |  |
| oe_lof | Observed over expected ratio for pLoF variants in transcript (obs_lof divided by exp_lof) |  |
| oe_lof_lower | Lower bound of 90% confidence interval for o/e ratio for pLoF variants | |
| syn_z | Z score for synonymous variants in gene. Higher (more positive) Z scores | |
| lof_z | Z score for pLoF variants in gene. Higher (more positive) Z scores indicate that the transcript is more intolerant of variation (more constrained) |  |
| oe_lof_upper_rank | Transcriptï¿½s rank of LOEUF value compared to all transcripts (lower values indicate more constrained) |  |
| n_sites | Number of distinct pLoF variant sites in the transcript | |
| classic_caf | Sum of allele frequencies of pLoFs in the transcript | |
| max_af | Maximum allele frequency of any pLoF in the transcript | |
| no_lofs | The number of individuals with no observed pLoF variants in the transcript | |
| obs_hom_lof | The number of individuals with at least one observed homozygous pLoF in the transcript |  |
| exac_obs_lof | Number of observed pLoF variants in gene in ExAC | |
| exac_exp_lof | Number of expected pLoF variants in gene in ExAC | |
| exac_oe_lof | Observed to expected ratio of pLoF variants in ExAC | |
| GDI | Gene Damage Index | https://lab.rockefeller.edu/casanova/GDI |
| ni | The neutrality index | Bayrak et. al |
| clarkd | The clark distance | Biopython |
| cds_len | The coding sequence length | biomaRt R package (biomart:2.52.0, R:4.1.2) |
| betweenness | The Betweenness centrality of the gene in the homo-sapiens protein-protein interaction network (BioGRID) | |
| interior | the percentage of residues having RSA <= 0.25 in a protein structure, referring to residues buried in the interior | Internal Dataset |
| ionic_n | The percentage of ionic interactions in the canonical protein AF structure with cut-off < 5 Angstroms | |
| catpi_n | The percentage of Cation-PI interactions in the canonical protein AF structure with cut-off < 5 Angstroms | |
| aroro_n | The percentage of aromatic-aromatic interactions in the canonical protein AF structure with cut-off < 5 Angstroms | |
| arosul_n | The percentage of aromatic-sulphide interactions in the canonical protein AF structure with cut-off < 5 Angstroms | |
| disulphide_n | The percentage of disulphide interactions in the canonical protein AF structure with cut-off < 5 Angstroms | |
| mmhbonds_n | The percentage of main-main chain hydrogen bonds interactions in the canonical protein AF structure with cut-off < 5 Angstroms | |
| mshbonds_n | The percentage of main-side chain hydrogen bonds interactions in the canonical protein AF structure with cut-off < 5 Angstroms | |
| sshbonds_n | The percentage of side-side chain hydrogen bonds interactions in the canonical protein AF structure with cut-off < 5 Angstroms | |
| AAAvgClustering | In network science, it is also known as the mean clustering coefficient. It is a network global clustering metric that measures the tendency of a given network to form separate clusters. | |
| MW | The Molecular Weight of the protein | Biopython |
| rsa_molecule | It is the Relative Surface Area of the molecule. It is defined as the ratio between the Moleculeï¿½s SASA divided by a theoretical SASA of the same sequence (Shrake & Rupley, 1973). | |
| helices_ratio | The percentage of helices in the canonical protein AF structure | Biopython |
| sheets_ratio | The percentage of sheets in the canonical protein AF structure | |
| avgevorate | the evolutionary rate of the differences in sequence at each residue position of each protein given multiple sequence alignment between 46 different species divided by the total evolutionary time of a known human phylogenetic tree (Kumar et al., 2009) | Kumar et.al , 2009 |
| ages | The estimated gene age calculated from phylogenetic analysis for closely related species (Kumar et al.,2009) | Kumar et.al , 2009 |
| rvis | It stands for Residual Variation Intolerance score. It is an intolerance score calculated on 6503 whole exome sequences from the NHLBI Exome sequencing project (ESP) and it relatively quantifies whether a gene harbors more or less functional variants than expected based on a background neutral variation model (Petrovski et al., 2013). Positive scores indicate the presence of more functional variants in a gene whereas negative scores indicate an intolerance of functional variations. We used RVIS latest release (v.4) which is based on Exac V.2 (Petrovski et al., 2013) | Petrovski et. al, 2003 |
| n_paralogs | The number of protein paralogs extracted from Ensembl Biomart(version 2.52.0) | biomaRt R package (biomart:2.52.0, R:4.1.2) |
| Disulfide_bond | The percentage of disulphide bonds in a protein divided by the number of non-covalent interactions within a cutoff < 5 Angstroms | Biopython |
| cosmis | COntact Set MISsense tolerance, It quantifies the tolerance of protein sites to genetic variation | Bian Li et. Al, 2022 |
| cossyn | contact set synonymous tolerance score quantified through synonymous variants | |
| gnomAD_distance | The numerator of Extent of Disease clustering (EDC) metric calculated from gnomAD benign mutations in a gene | Gerasimavicius et al., 2022 |
| clustering | The Extent of disease clustering (EDC) | |
| smc_log10_ci_low | The lower bound of the confidence interval for logarithmic Monte Carlo Approximate Bayesian Computation (ABC-SMC) score | Agarwal et.al , 2022 |
| smc_log10_map | The estimated mean maximum a posteriori for Monte Carlo Approximate Bayesian Computation (ABC-SMC) score | |
| is_homomer | Binary variable indicating if the protein is homomeric | Biopython |
| is_heteromer | Binary variable indicating if the protein is heteromeric | |
| GO:0005739 | A set of Gene Ontology Terms that were significantly over-represented between better predicted genes and badly predicted genes based on the median of AUROC calcualted from the entire missense mutations dataset | GO Ontology, PantherDB |
| GO:0000122 | |  |
| GO:0006355 | |  |
| GO:0006357 | |  |
| GO:0032502 | |  |
| GO:0032501 | |  |
| GO:1901605 | |  |
| GO:0006520 | |  |
| GO:0019752 | |  |
| GO:0044281 | |  |
| GO:0140110 | |  |
| GO:0001067 | |  |
| ndomains | The number of domains in a protein calculated from pFAM database | pFAM Database |
| max_domain_len | The maximum domain length percentage calculated from pFAM database | |
| evolutionary_rate | for each gene, we calculated the natural log of the mean of the difference in sequence among the selected species (1 - percent identities) divided by double the evolutionary unit time (Agarwal et al., n.d.; Kumar et al., 2009). | Ensembl Compara and BioPython |
| divergence_score | Defined as the standard deviation of the percent identities among different species per gene | |
| evo_diff_range | The difference between the maximum identity and the minimum identity among gene homologs across 13 evolutionarily related species in Ensembl Compara Database | |
| mean_plddt | The average predicted Local Distance Difference Test (pLDDT) for all predicted residues in a protein | Biopython |
| benign_odds | The odds of benign mutations in a gene | |
| pLI_intolerant | A binary variable indicating whether the gene has pLI > 0.9 | Derived from gnomAD |
| exac_pLI_intolerant | A binary variable indicating whether the gene has exac_pLI > 0.9 | |
| pLI_tolerant | A binary variable indicating whether the gene has pLI <= 0.9 | |
| exac_pLI_tolerant | A binary variable indicating whether the gene has pLI <= 0.9 | |
| protein_len | the length of protein sequence | uniprots KB |
| domain_ratio | The average domain length in a protein divided by protein length | pFAM Database |
| ddG_fold | It is calculated as the average protein stability metric (??G) for each proteinï¿½s known putatively benign variants in gnomAD (version 2.1) using their predicted structures from Alphafold V2 | Biopython, Sciki-learn, calculated from our internal pipeline |
| hm_dn_ds | The human nonsynonymous to synonymous ratio | Biopython |
| localization_CellMembrane | A set of binary variables denoting the localization of the protein intracellularly derived from Protein Atlas | Human Protein Atlas |
| localization_Cytoplasm | | |
| localization_Endoplasmicreticulum | | |
| localization_Extracellular | | |
| localization_GolgiApparatus | | |
| localization_Lysosome/Vacuole | | |
| localization_Mitochondrion | | |
| localization_Nucleus | | |
| localization_Peroxisome | | |
| localization_Plastid | |  |
| localization_Membranebound | | |
| plddt_disorder | The ratio of proteinï¿½s disorder explained by residues having pLDDT < 50 across all the protein. | Alphafold Models and Biopython |
| vep_gnomad_mean | This feature is a placeholder which represents the average raw VEP score for all benign mutations per gene for each VEP, corrected for inverted VEPs. For instance, for ESM-1v, this feature will represent the average ESM-1v raw scores for all benign mutations per gene. | |
| mpossible_mutations | This feature is a placeholder which represents the mean raw score for each VEP calculated for all possible predicted mutations in a protein  similar to vep_gnomad_mean. |  |
| shet | Posterior mean for shet (https://zenodo.org/record/7939768/files/s_het_estimates.genebayes.tsv, md5:b4add050ea9ab507e9e0fed338069ccc) | https://zenodo.org/record/7939768/files/s_het_estimates.genebayes.tsv |
| efx_abs | Median ratio of ESM-1v (https://doi.org/10.1101/2021.07.09.450648) and the absolute value of FoldX (https://foldxsuite.crg.eu/) calculated change in Gibbs-free energy of folding (Î”Î”G) of all missense mutations based on possible amino acid substitutions. | (https://doi.org/10.1101/2021.07.09.450648) and (https://foldxsuite.crg.eu/) |
| efx_raw | As above but with raw (non-absolute-value) Î”Î”G scores. | |
| ct | Cotranslational assembly annotations from Bertolini et al., 2021. (doi: 10.1126/science.abc7151). | Bertolini et al., 2021. |
| pct_buried | Fraction of residues with less than 25% relative solvent accessible surface area in the AlphaFold predicted structure. | |
| pLOF | Predicted LOF probability at the gene-level - Badonyi and Marsh, 2024 | |
| pGOF | Predicted GOF probability at the gene-level - Badonyi and Marsh, 2024 | |
| pDN | Predicted DN probability at the gene-level - Badonyi and Marsh, 2024 | |

**Table S2: Overall of ranking of feature importance across random forest models for all VEPs.**

| feature | rankmean |
| --- | --- |
| Multicellular Organismal Process (GO:0032501) | 1.971429 |
| ddG_fold | 5.914286 |
| Developmental Process (GO:0032502) | 8.771429 |
| vep_gnomad_mean | 11.57143 |
| gnomAD_distance | 11.68571 |
| plddt_disorder | 14.85714 |
| betweenness | 15.51429 |
| nTPM | 17.51429 |
| shet | 17.91429 |
| exac_oe_lof | 19.57143 |
| mean_plddt | 21.05714 |
| n_sites | 21.08571 |
| pLOF | 21.57143 |
| efx_raw | 22.02857 |
| max_af | 26.17143 |
| clustering | 27.42857 |
| lof_z | 28.71429 |
| efx_abs | 29.08571 |
| ni | 29.28571 |
| benign_odds | 29.71429 |
| oe_lof_upper_rank | 29.94286 |
| exp_mis | 30.17143 |
| divergence_score | 30.57143 |
| oe_lof_lower | 31.37143 |
| Cytosol (GO:0005829) | 31.97143 |
| smc_log10_map | 32.31429 |
| evolutionary_rate | 32.45714 |
| classic_caf | 33.25714 |
| pGOF | 33.45714 |
| oe_mis | 34.11429 |
| oe_lof | 34.17143 |
| evo_diff_range | 34.42857 |
| pDN | 35.05714 |
| obs_lof | 35.54286 |
| hm_dn_ds | 35.97143 |
| rsa_molecule | 36 |
| clarkd | 36.4 |
| AAAvgClustering | 36.91429 |
| GDI | 38.51429 |
| smc_log10_ci_low | 40.02857 |
| possible_mis | 40.17143 |
| domain_ratio | 40.45714 |
| rvis | 41.51429 |
| avgevorate | 41.6 |
| transcript_count | 42.42857 |
| sshbonds_n | 43.14286 |
| cossyn | 43.62857 |
| ionic_n | 43.82857 |
| possible_lof | 44.2 |
| interior | 44.45714 |
| pct_buried | 44.62857 |
| obs_mis | 45.54286 |
| no_lofs | 46.4 |
| exac_exp_lof | 46.54286 |
| helices_ratio | 46.65714 |
| protein_len | 48.08571 |
| cosmis | 48.62857 |
| sheets_ratio | 49 |
| mshbonds_n | 50 |
| pRec | 52.25714 |
| n_paralogs | 53 |
| arosul_n | 53.17143 |
| is_homomer | 56.48571 |
| cds_len | 57.51429 |
| exac_obs_lof | 58.37143 |
| max_domain_len | 58.82857 |
| disulphide_n | 60.05714 |
| Regulation of DNA-templated transcription (GO:0006355) | 61.14286 |
| catpi_n | 62.11429 |
| GO:0044281 | 62.85714 |
| ages | 63.8 |
| GO:0005739 | 65 |
| MW | 66.4 |
| ndomains | 67.97143 |
| aroro_n | 70.68571 |
| mu_lof | 71.6 |
| localization_Cytoplasm | 73.91429 |
| GO:0006357 | 77.4 |
| localization_Membranebound | 77.71429 |
| is_heteromer | 79.8 |
| ct | 81.05714 |
| exac_pLI_intolerant | 81.08571 |
| localization_Endoplasmicreticulum | 81.28571 |
| localization_Nucleus | 84.25714 |
| localization_CellMembrane | 85.02857 |
| localization_Mitochondrion | 85.48571 |
| exac_pLI_tolerant | 86.51429 |
| obs_hom_lof | 86.65714 |
| GO:0000122 | 87.45714 |
| localization_Extracellular | 87.85714 |
| GO:0140110 | 88.37143 |
| pLI_intolerant | 89.8 |
| GO:0001067 | 90.47143 |
| pLI_tolerant | 91.08571 |
| GO:0019752 | 92.37143 |
| localization_GolgiApparatus | 93.57143 |
| localization_Lysosome/Vacuole | 97.2 |
| GO:0006520 | 97.94286 |
| GO:1901605 | 99.31429 |
| localization_Peroxisome | 100.5286 |
| Disulfide_bond | 102.5143 |
| Formation_of_an_isopeptide_bond | 102.5143 |
| localization_Plastid | 102.5143 |
| mmhbonds_n | 102.5143 |
| ntissues | 102.5143 |
